# Bidirectional associations between periodontal disease and systemic diseases: a nationwide population-based study in Korea

**DOI:** 10.1038/s41598-023-41009-4

**Published:** 2023-08-28

**Authors:** Salma Nabila, Jaesung Choi, Ji-Eun Kim, Seokyung Hahn, In-Kyung Hwang, Tae-Il Kim, Hee-Kyung Park, Ji-Yeob Choi

**Affiliations:** 1 Department of Biomedical Sciences, Seoul National University Graduate School, 103 Daehak-ro, Jongno-gu, Seoul, 110-799 Korea; 2 BK21plus Biomedical Science Project, Seoul National University College of Medicine, Seoul, Korea; 3 Institute of Health Policy and Management, Seoul National University Medical Research Center, Seoul, Korea; 4 Department of Epidemiology and Cancer Control, St. Jude Children’s Research Hospital, Memphis, TN USA; 5 Division of Medical Statistics, Medical Research Collaborating Center, Seoul National University Hospital, Seoul, Korea; 6 Department of Medicine, Seoul National University College of Medicine, Seoul, Korea; 7 Department of Periodontology, Dental Research Institute, Seoul National University Dental Hospital, Seoul National University School of Dentistry, Seoul, Korea; 8 Department of Oral Medicine and Oral Diagnosis, Dental Research Institute, Seoul National University Dental Hospital, Seoul National University School of Dentistry, 101 Daehak-ro, Jongno-gu, Seoul, 110-749 Korea; 9 Cancer Research Institute, Seoul National University, Seoul, Korea

**Keywords:** Risk factors, Epidemiology, Preventive dentistry, Cardiovascular diseases, Dental diseases, Diabetes

## Abstract

To evaluate the associations of periodontal disease (PD) with systemic diseases, including diabetes mellitus (DM) and cardiovascular disease (CVD), as well as the reciprocal association. The CVD included the cases of coronary heart disease and heart failure. A prospective study was conducted from 2007 to 2019 using linked data from three databases in Korea. Three separate study groups were formed to individually determine the risks of PD (n = 10,533), DM (n = 14,523) and CVD (n = 14,315). All diseases were confirmed based on physicians’ diagnoses using medical records and self-reports. Cox proportional hazard regression was applied with 95% confidence intervals (CIs) to obtain hazard ratios (HRs). PD was significantly associated with an elevated risk of DM (HR [95% CI]: 1.22 [1.07–1.39]) after full adjustment for age, sex, lifestyle factors, body mass index, dental behaviour and CVD. PD was also found to increase the risk of CVD (1.27 [1.03–1.57]), whereas CVD increased the risk of PD (1.20 [1.09–1.32]) after full adjustment for other covariates including DM. This study found a bidirectional association between PD and CVD, as well as a positive association of PD with DM.

## Introduction

Systemic diseases such as diabetes mellitus (DM) and cardiovascular disease (CVD) have been widely acknowledged as a significant problem, with the number of affected people increasing globally, including in Korea^1,2^. A recent national survey report revealed that the prevalence of DM among Korean adults has increased over the last few years^1^. Likewise, according to a 2020 analysis of nationwide data, the trends of mortality and hospitalisation caused by CVD have increased over the last 10 years^2^. The global burden of oral disease has also now been recognised, with 20–50% of the population in various countries being affected depending on the criteria used for disease classification^3^. Particularly regarding Korea, recent statistics showed that oral disease ranked first among the diseases receiving the most benefits from health care insurance^4^.

The association between periodontal and systemic diseases, including DM and CVD, is widely recognised^4,5^. Robust evidence from previous prospective studies has shown an elevated risk of DM among individuals with periodontal disease (PD)^6–10^. A previous systematic review reported that PD appeared to increase the risk of DM by 26%^6^, while another study that considered only severe PD reported a 53% increased risk^7^. PD has also been reported as a risk factor for CVD based on consistent evidence from prior studies^3,11^. In a meta-analysis of 32 longitudinal studies, the risk of CVD was found to be 1.2 times higher in individuals with PD^12^.

The bidirectional association between DM and PD has gained attention since prospective population-based studies not only found that PD is a risk factor for DM^8–10^ but also that there is a reciprocal association between the two^8,13–15^. A possible explanation for this association is the inflammatory process involved in the progression of both diseases^16,17^. However, other prospective studies provided conflicting evidence, wherein no association was observed^18–20^. Furthermore, longitudinal prospective studies exploring both associations in the same population are scarce. Morita et al. conducted such a study and reported the presence of a bidirectional association^8^; however, another study by Alshihayb et al. did not reveal any association between the two diseases and suggested the possible effect of confounders^19^. Although the reasons underlying this contrast are not clear, possibilities include the large difference in sample size and differences in the regions where the studies were conducted. Accordingly, further population-based studies are needed to clarify the two-way association between PD and DM.

Apart from DM, CVD is also reported to increase the risk of PD^11^ through several possible mechanisms, including the influence of periodontal bacteria in atheroma lesions and in the development of atherosclerosis, elevations in inflammatory markers and fibrinogen, cross-reactivity between antibodies from periodontal pathogens and antigens in cardiovascular tissue, and the presence of hyper-response in reactive oxygen species in patients with periodontitis^11^. While the body of evidence regarding the association between periodontal and cardiovascular diseases is well established, the reverse association has not been adequately explored^11^. Therefore, further study on this topic is warranted.

Clarifying the bidirectional relationships between PD and DM as well as CVD may be important for preventing these diseases. Especially in Korea, there was no population-based studies have evaluated these relationships. Therefore, we conducted a prospective study with the following main objectives: (1) to determine the bidirectional association between PD and DM and (2) to determine the bidirectional association between PD and CVD in the same population using several linked databases in Korea.

## Methods

### Study design and population

The source population for this study comprised the participants of the Korean National Health and Nutrition Examination Survey from 2007 to 2015 (KNHANES IV–VI). Detailed information on this study has been given previously^21,22^. Briefly, the KNHANES is a nationwide surveillance system used to assess data from individuals aged more than 1 year by conducting health interviews, health examinations and nutrition surveys. From the database of this survey, baseline data on sociodemographic and lifestyle factors, body mass index (BMI), biomarkers, dental behaviours, and self-reported disease history were obtained. To obtain outcome information, this database was linked to two other administrative claims databases in South Korea, including the Health Insurance Review and Assessment Service (HIRA) 2007–2019 and the National Health Insurance Service-National Health Information Database (NHIS-NHID) 2007–2015. The HIRA is a database containing detailed medical records of Korean residents who visited pharmacies, hospitals or physicians^23^, while the NHIS-NHID is a database organised by the Korean National Health Insurance Service that contains information on the health care utilisation, health screening, sociodemographics and mortality of the Korean population^24^. By combining the three databases, information on participants’ medical records from the HIRA and death information from the NHID were obtained. The Korea Health Information Service organised and merged the three databases.

The baseline or index period of this study was set from 2009 to 2015, and the participants were followed up until 2019. A washout period from 2007 to 2008 was applied to minimise the risk of reverse causality of the outcome. For better understanding, the scheme of this study is detailed in Supplementary Fig. 1.

**Figure 1:**
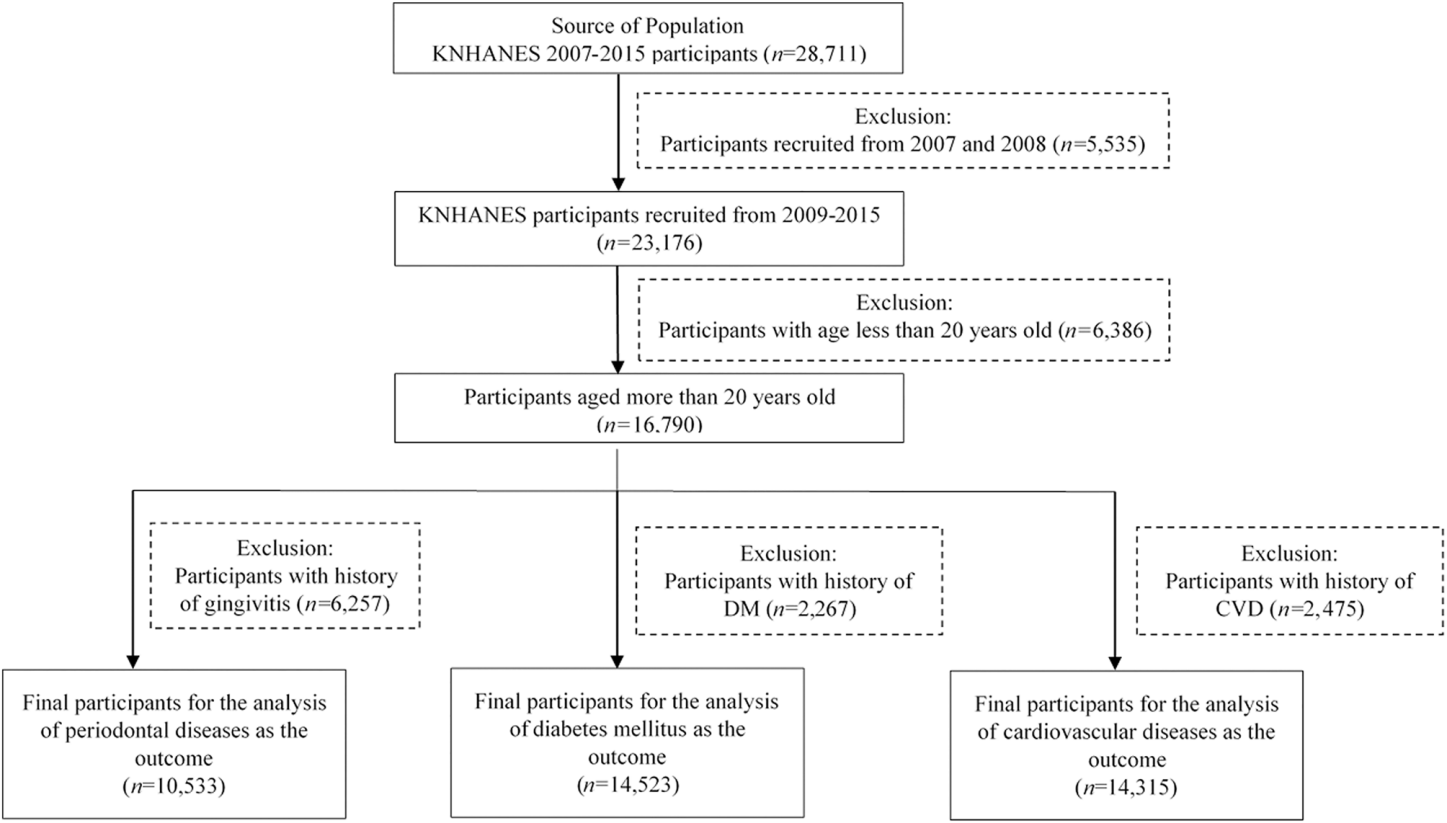
Flowchart of participant selection.

To evaluate the association between PD and DM as well as CVD, three schemes were used: (1) the analysis of PD as the outcome, (2) the analysis of DM as the outcome, and (3) the analysis of CVD as the outcome. Among all the participants of the KNHANES 2007–2015 (n = 28,711), 535 participants recruited during the washout period (2007–2008) and 6386 participants aged less than 20 years were excluded. Thus, 16,790 participants remained in the study. Three dataset groups were formed. For the first, participants with a history of PD before the index period (n = 6257) were excluded, leaving 10,533 eligible participants for the analysis. For the second, participants with a history of diabetes (n = 2267) were excluded, leaving 14,523 participants. For the third, participants with a history of CVD (n = 2475) were excluded; thus, 14,315 participants were included. The detailed flow of study participant selection is shown in Fig. 1.

### Ascertainment of periodontal disease

PD in the present study included gingivitis and periodontitis. Information on PD was obtained from the HIRA and identified according to the International Classification of Diseases 10 (ICD-10). The incidence was ascertained if the participant had visited a physician at least twice within a month with the periodontitis diagnostic code K05 or one of the following health claim-of-treatment codes was used: U2240, U1010, U1020, U1051, U1052, U1071, U1072, U1081, U1082, U4412, U4413 or U4414. Details regarding disease identification are provided in Supplementary Table 1.

### Ascertainment of diabetes mellitus

DM cases as exposure were ascertained using the ICD-10 codes from the medical record data in the HIRA database before the index period and self-reported disease history from the KNHANES. Participants were considered to have DM if there was a medical record of prescription of diabetes medication or the provision of ICD-10 codes E11, E12, E13 or E14 at least twice within 1 year in the medical record before the index period or if the participant reported a history of being diagnosed with diabetes by a physician in the KNHANES data. Furthermore, DM was ascertained based only on the medical record data in the HIRA during the follow-up period. The details of disease identification are provided in Supplementary Table 1 and Supplementary Fig. 1.

### Ascertainment of cardiovascular disease

In this study, CVD included cases of coronary heart disease and heart failure. CVD was ascertained using the ICD-10 codes from the medical record data in the HIRA database and self-reported disease history in the KNHANES data. Coronary heart disease was confirmed based on medical records indicating hospitalisation for at least 2 days because of the disease codes I20–I25 before the index period, the self-reported diagnosis of angina, or the diagnosis of myocardial infarction by a physician. Participants were considered to have heart failure if there was a medical record indicating hospitalisation for at least 2 days because of the disease code I50 before the index period. Furthermore, CVD was ascertained using the medical record data from the HIRA database during the follow-up period. The details of disease identification are provided in Supplementary Table 1 and Supplementary Fig. 1.

### Covariates

To address potential bias from potential covariates, some other variables were included in this study, such as sociodemographic factors (sex, income level, age and residential area), lifestyle factors (smoking status, smoking pack-years, alcohol consumption and physical activity), BMI, biomarkers (aspartate transaminase (AST), alanine aminotransferase (ALT), fasting plasma glucose (FPG), cholesterol and blood pressure), dental behaviour (brushing frequency, use of dental floss, use of an interdental brush and use of rinsing solution) and oral health at baseline. A history of other diseases (tooth decay, cancer, cerebrovascular diseases, hypertension, hyperlipidaemia, arthritis, rheumatic diseases, gastrointestinal diseases and infectious diseases) was also included. The inclusion of the covariates was based on previous studies reporting associations between some of these variables and PD^25–27^, DM^1,28,29^, and CVD^30,31^.

Smoking status was classified as never smoker, former smoker or current smoker based on participant reports regarding current smoking habits. Smoking pack-years were calculated by multiplying the number of cigarette packs smoked per day by the total number of smoking years, and participants were categorised based on their total smoking pack-years as light smokers (0.1–20 pack-years), moderate smokers (20.1–40 pack-years) and heavy smokers (> 40 pack-years)^32^. Participants were divided into non-drinkers and drinkers according to their alcohol consumption habits. Furthermore, participants were grouped into underweight (< 18.5 kg/m^2^), normal (18.5–22.9 kg/m^2^), overweight (23–24.9 kg/m^2^) and obese (≥ 25 kg/m^2^) BMI categories following the recommended classification for Koreans^33^.

Oral health at baseline was represented by the score of the Decayed, Missing, and Filled Teeth (DMFT) index. The DMFT index is one of the indices used to assess oral health based on the number of decayed, missing and filled teeth^34^. Disease history was identified using ICD-10 codes and self-reports. The ascertainment of disease history is presented in Supplementary Table 1. A separate category was allocated for missing information on each covariate and included in the analysis.

### Statistical analysis

Cox proportional hazard regression with 95% confidence intervals (CIs) was applied to obtain the hazard ratios (HRs) of PD, DM and CVD. The time variable was defined as the duration from the index date to the diagnosis of outcome, death or end of follow-up, whichever came first. Participants who did not have PD or died before the endpoint of follow-up were treated as censors. Similarly, in the other groups, those who did not have DM or CVD or died before the end of follow-up were treated as censors.

Five models were assessed in the main analysis: model 1 was adjusted for age and sex; model 2 was adjusted for age, sex, lifestyle factors and BMI; model 3 was adjusted for age, sex, lifestyle factors, BMI and dental behaviour; model 4 was adjusted for age, sex, lifestyle factors, BMI, DM and CVD; and model 5 was adjusted for age, sex, lifestyle factors, BMI, dental behaviour, DM and CVD. A stratified analysis by sex along with a heterogeneity test using Cochran’s Q test and the I^2^ test was also performed^35^. All analyses were performed using SAS statistical software (version 9.4; SAS Institute, Cary, NC, USA).

### Ethics approval and consent to participate

This study was approved by the Institutional Review Board of the Seoul National University Hospital, Seoul, Korea (IRB No: E-1911-054-1078).

## Results

Among the participants in the first group, 4531 (43.02%) developed PD after a median follow-up of 5 years (mean = 5.4 years). Participants with a higher income (HR: 1.09 [95% CI: 1.00–1.88]), older age (HR: 1.84 [95% CI: 1.69–2.00]) and a higher BMI (HR: 1.19 [95% CI: 1.10–1.27]) at baseline were more likely to have PD. Furthermore, of all the dental behaviours, higher tooth-brushing frequency, the use of mouth rinsing solution, and a higher DMFT index score were found to increase the PD risk in all models (HR: 1.17 [95% CI: 1.02–1.34], 1.21 [95% CI: 1.11–1.32] and 1.20 [95% CI: 1.11–1.29], respectively). The presence of any disease history was also positively associated with a higher risk of PD (Table 1).

**Table 1:** Baseline characteristics and hazard ratio for periodontal disease.

Variables	Total	Periodontal disease	
	N (%)	Event (%)	HR (95% CI)
	10,533	4531 (43.02)	
Sociodemographic factors			
Sex			
Men	3223 (30.60)	1314 (29.00)	Reference
Women	7310 (69.40)	3217 (71.00)	1.01 (0.94–1.08)
Income level^1^			
< 200	3590 (34.08)	1532 (33.81)	Reference
200–399	3386 (32.15)	1491 (32.91)	1.13 (0.05–1.23)
≥ 400	3557 (33.77)	1508 (33.28)	1.09 (1.00–1.88)
Age			
20–39	1336 (12.68)	488 (10.77)	Reference
40–59	2370 (22.50)	850 (18.76)	1.55 (1.44–1.67)
≥ 60	2066 (19.61)	904 (19.95)	1.84 (1.69–2.00)
Residential area			
Urban	8163 (77.50)	3526 (77.82)	Reference
Rural	2370 (22.50)	1005 (22.18)	0.96 (0.89–1.04)
Lifestyle factors and BMI			
Smoking status			
Never smokers	6867 (67.96)	3002 (68.04)	Reference
Former smokers	1817 (17.98)	837 (18.97)	1.08 (0.98–1.19)
Current smokers	1420 (14.05)	573 (12.99)	1.00 (0.90–1.12)
Smoking pack-year			
Never smokers	6867 (71.26)	3091 (72.87)	Reference
Light smokers	1919 (19.91)	770 (18.15)	1.02 (0.93–1.13)
Moderate smokers	558 (5.79)	257 (6.06)	1.04 (0.89–1.22)
Heavy smokers	292 (3.03)	124 (2.92)	1.11 (0.89–1.37)
Missing	897	299	
Alcohol consumption			
Non-drinker	3056 (30.30)	1354 (30.75)	Reference
Drinker	7030 (69.70)	3050 (69.26)	1.02 (0.95–1.09)
Physical activity			
No exercise	4382 (53.26)	2015 (51.87)	Reference
< 150 min/wk	979 (11.90)	467 (12.02)	1.03 (0.93–1.14)
≥ 150 min/wk	2867 (34.84)	1403 (36.11)	1.04 (0.97–1.12)
Missing	2305	646	
BMI (kg/m^2^)			
< 18.5	487 (4.64)	168 (3.71)	0.95 (0.81–1.12)
18.5–22.9	4524 (43.10)	1809 (40.00)	Reference
23–24.9	2423 (23.08)	1120 (24.76)	1.14 (1.06–1.23)
≥ 25	3062 (29.17)	1426 (31.53)	1.19 (1.10–1.27)
Biomarkers^2^			
AST			
Normal	9432 (3.41)	4146 (96.82)	Reference
High	333 (96.59)	136 (3.18)	0.98 (0.82–1.18)
Missing	768	249	
ALT			
Normal	9215 (94.37)	4044 (94.44)	Reference
High	550 (5.63)	238 (5.56)	1.01 (0.88–1.15)
Missing	768	249	
FPG			
< 100	7237 (74.16)	3160 (73.85)	Reference
100–125	1908 (19.55)	831 (19.42)	0.98 (0.90–1.06)
≥ 126	614 (6.29)	288 (6.73)	1.08 (0.95–1.23)
Missing	774	252	
Cholesterol			
< 200	6395 (65.49)	2722 (63.57)	Reference
200–239	2582 (26.44)	1185 (27.67)	1.03 (0.96–1.10)
≥ 240	788 (8.07)	375 (8.76)	1.06 (0.95–1.19)
Missing	768	249	
Blood pressure			
Normal	7831 (74.61)	3288 (72.71)	Reference
Pre-hypertension	2180 (20.77)	1009 (22.31)	1.04 (0.97–1.13)
Hypertension	485 (4.62)	225 (4.98)	1.00 (0.87–1.15)
Dental behavior			
Brushing frequency			
≤ 1	1707 (16.21)	606 (13.37)	Reference
2	4000 (37.98)	1856 (40.96)	1.32 (1.18–1.46)
3	3561 (33.81)	1556 (34.34)	1.25 (1.12–1.40)
≥ 4	1265 (11.32)	513 (11.32)	1.17 (1.02–1.34)
Dental floss			
No	8359 (82.25)	3660 (82.84)	Reference
Yes	1804 (17.75)	758 (17.16)	1.01 (0.93–1.10)
Interdental brush			
No	9002 (88.58)	3896 (88.18)	Reference
Yes	1161 (11.42)	522 (11.82)	1.03 (0.82–1.29)
Mouth rinsing solution			
No	8854 (87.12)	3803 (86.08)	Reference
Yes	1309 (12.88)	615 (13.92)	1.21 (1.11–1.32)
DMFT			
≤ 4	3660 (36.39)	1439 (32.88)	Reference
8–5	2855 (28.39)	1399 (31.97)	1.37 (1.27–1.48)
> 8	3543 (35.23)	1538 (35.15)	1.20 (1.11–1.29)
Disease history (yes versus no)			
Tooth decay	3118 (29.60)	1648 (36.37)	1.50 (1.40–1.60)
Cancer	1145 (10.87)	579 (12.78)	1.16 (1.06–1.27)
Cerebrovascular diseases	405 (3.85)	186 (4.11)	1.17 (1.00–1.38)
Hypertension	2987 (28.36)	1448 (31.96)	1.20 (1.11–1.29)
Hyperlipidemia	1285 (12.2)	699 (15.43)	1.37 (1.26–1.50)
Arthritis and rheumatic	3253 (30.88)	1662 (36.68)	1.36 (1.26–1.50)
Osteoporosis	986 (9.36)	497 (10.97)	1.11 (1.00–1.24)
Gastrointestinal	6994 (66.4)	3413 (75.33)	1.72 (1.61–1.85)
Infectious diseases	4791 (45.49)	2312 (51.03)	1.38 (1.30–1.47)

*HR* hazard ratio adjusted for age, sex, and dental behavior, *BMI* body mass index, *AST* aspartate transaminase, *ALT* alanine transaminase, *FPG* fasting blood glucose, *DMFT* Decayed, Missing, and Filled Teeth index.

^1^Income is in Korean 10,000 won.

^2^AST and ALT levels were considered high if the value was > 40 IU/L; blood pressure was grouped into normal (systolic < 120 and diastolic < 80 mmHg), pre-hypertension (systolic 120–139 and diastolic 80–89 mmHg), and hypertension (systolic ≥ 140 or diastolic ≥ 90 mmHg).

Supplementary Table 2 shows the HRs for DM. After a median follow-up of 7 years (mean = 6.9 years), 1151 (7.93%) of the participants in the second group developed DM. After fully adjusting for other covariates, older age groups were found to have an increased risk of DM (HR: 5.42 [95% CI: 4.28–6.86]). Regarding potential lifestyle risk factors, the risk of DM was found to be elevated for heavy smokers (HR: 1.64 [95% CI: 1.03–2.62]). Furthermore, higher risk was also observed for participants with a high BMI (HR: 2.35 [95% CI: 2.02–2.73]), high AST (HR: 1.96 [95% CI: 1.52–2.53]), high ALT (HR: 1.99 [95% CI: 1.61–2.46]), high FPG (HR: 3.17 [95% CI: 2.75–3.66] for 100–125 mg/dL and HR: 13.58 [95% CI: 11.13–16.56] for ≥ 126 mg/dL), cholesterol ≥ 240 mg/dL (HR: 1.44 [95% CI: 1.19–1.74]) and high blood pressure (HR: 1.41 [95% CI: 1.21–1.76]). The presence of any disease history (except cerebrovascular disease) was also found to be significantly associated with a higher risk of DM.

The baseline characteristics and HRs for CVD are presented in Supplementary Table 3. After a median follow-up of 7 years (mean = 7.1 years), 509 (3.56%) participants had CVD. An increased risk of CVD was observed for participants with older age (HR: 15.02 [95% CI: 9.24–24.40]), BMI ≥ 25 kg/m^2^ (HR: 2.00 [95% CI: 1.57–2.55]) and high blood pressure (HR: 3.08 [95% CI: 2.29–4.16]). Furthermore, a history of tooth decay, DM, cancer, hypertension, hyperlipidaemia, rheumatoid arthritis, osteoporosis, and gastrointestinal and infectious diseases was observed to be associated with an elevated risk of CVD.

Supplementary Table 4 presents an additional analysis of the association between BMI and the risk of PD, DM and CVD using other categories. The results showed that the risk of systemic diseases was even higher after dividing the obese category into 25–27.4 kg/m^2^ and ≥ 27.5 kg/m^2^ subcategories as well as 25–29.9 kg/m^2^ and ≥ 30 kg/m^2^ subcategories. The HRs for DM were 1.98 [95% CI: 1.48–2.64] and 1.94 [95% CI: 1.61–2.35] for BMI ≥ 27.5 kg/m^2^ and ≥ 30 kg/m^2^, respectively, while the HRs for CVD were 2.09 [95% CI: 1.21–3.60] and 2.24 [95% CI: 1.64–3.07] for BMI ≥ 27.5 kg/m^2^ and ≥ 30 kg/m^2^, respectively.

The two-way associations between PD and chronic diseases are shown in Table 2. In the first group, 514 (11.34%) participants with DM and 615 (51.99%) participants with CVD developed PD. Furthermore, among the participants with PD in the second group, 503 (43.70%) were diagnosed with DM, and among those in the third group, 514 (11.34%) were diagnosed with CVD during follow-up. A positive association between PD and DM risk was found in all models, with HR values of 1.23 [95% CI: 1.08–1.39], 1.25 [95% CI: 1.10–1.42], 1.25 [95% CI: 1.10–1.42], 1.22 [95% CI: 1.08–1.39] and 1.22 [95% CI: 1.07–1.39] for models 1 to 5, respectively. Regarding the reverse association, PD risk was found to increase in individuals with DM compared to those without DM after adjustment in models 1, 2 and 3 (HR: 1.15 [95% CI: 1.03–1.27], 1.13 [95% CI: 1.02–1.25] and 1.13 [95% CI: 1.02–1.25], respectively). However, in models 5 and 6, the association was not significant (HR: 1.09 [95% CI: 0.99–1.22] and 1.09 [95% CI: 0.99–1.22], respectively). The association between PD and an increased risk of CVD was also notable in all models (HR: 1.27 [95% CI: 1.03–1.56], 1.30 [95% CI: 1.05–1.59], 1.31 [95% CI: 1.06–1.61], 1.26 [95% CI: 1.02–1.55] and 1.27 [95% CI: 1.03–1.57], respectively). Similarly, regarding the reciprocal association, a higher risk of developing PD was found among participants with CVD at baseline compared to those without CVD (HR: 1.24 [95% CI: 1.13–1.37], 1.22 [95% CI: 1.11–1.35], 1.22 [95% CI: 1.10–1.34], 1.21 [95% CI: 1.09–1.33] and 1.20 [95% CI: 1.09–1.32] for models 1 to 5, respectively).

**Table 2:** Associations between periodontal disease and chronic diseases.

	Total	Events	HR (95% CI)				
	N (%)	N (%)	Model 1	Model 2	Model 3	Model 4	Model 5
Periodontal disease to diabetes mellitus							
Periodontal disease							
No	9434 (64.96)	648 (56.30)	Reference	Reference	Reference	Reference	Reference
Yes	5089 (35.04)	503 (43.70)	1.23 (1.08–1.39)	1.25 (1.10–1.42)	1.25 (1.10–1.42)	1.22 (1.08–1.39)	1.22 (1.07–1.39)
Diabetes mellitus to periodontal disease							
Diabetes mellitus							
No	9434 (89.57)	4017 (88.66)	Reference	Reference	Reference	Reference	Reference
Yes	1099 (10.43)	514 (11.34)	1.15 (1.03–1.27)	1.13 (1.02–1.25)	1.13 (1.02–1.25)	1.09 (0.99–1.22)	1.09 (0.99–1.22)
Periodontal disease to cardiovascular diseases							
Periodontal disease							
No	9350 (65.32)	296 (58.15)	Reference	Reference	Reference	Reference	Reference
Yes	4965 (34.68)	213 (41.85)	1.27 (1.03–1.56)	1.30 (1.05–1.59)	1.31 (1.06–1.61)	1.26 (1.02–1.55)	1.27 (1.03–1.57)
Cardiovascular disease to periodontal diseases							
Cardiovascular disease							
No	9350 (88.77)	3916 (88.77)	Reference	Reference	Reference	Reference	Reference
Yes	1183 (11.23)	615 (51.99)	1.24 (1.13–1.37)	1.22 (1.11–1.35)	1.22 (1.10–1.34)	1.21 (1.09–1.33)	1.20 (1.09–1.32)

Model 1: hazard ratio adjusted for age and sex.

Model 2: hazard ratio adjusted for age, sex, and lifestyle factors.

Model 3: hazard ratio adjusted for age, sex, lifestyle factors, and dental behavior.

Model 4: hazard ratio adjusted for age, sex, lifestyle factors, diabetes mellitus, and cardiovascular diseases.

Model 5: hazard ratio adjusted for age, sex, lifestyle factors, dental behavior, diabetes mellitus, and cardiovascular diseases.

Results from the stratified analysis by sex showed a substantial sex-based difference regarding the association between CVD and PD, which was significant only for women (Q test *p-*value = 0.02, I^2^ = 81). The complete results are presented in Table 3.

**Table 3:** Associations between periodontal disease and chronic diseases stratified by sex.

	Men			Women			Heterogeneity test	
	Total	Events		Total	Events			
	N (%)	N (%)	HR (95% CI)	N (%)	N (%)	HR (95% CI)	Q test *P–*value	I^2^ (%)
Periodontal disease to diabetes mellitus								
Periodontal diseases								
No	2816 (63.25)	202 (50.75)	Reference	6618 (65.71)	446 (59.23)	Reference		
Yes	1636 (36.75)	196 (49.25)	1.39 (1.11–1.74)	3453 (34.29)	307 (40.77)	1.15 (0.90–1.35)	0.18	45
Diabetes mellitus to periodontal disease								
Diabetes mellitus								
No	2816 (87.37)	1138 (86.61)	Reference	6618 (90.53)	2879 (89.49)	Reference		
Yes	407 (12.63)	176 (13.39)	1.07 (0.89–1.29)	692 (9.47)	338 (10.51)	1.11 (0.98–1.27)	0.75	0
Periodontal disease to cardiovascular disease								
Periodontal diseases								
No	2851 (63.97)	109 (61.24)	Reference	6499 (65.93)	187 (56.50)	Reference		
Yes	1606 (36.03)	69 (38.76)	1.21 (0.84–1.76)	3359 (34.07)	144 (43.50)	1.35 (1.05–1.74)	0.64	0
Cardiovascular disease to periodontal disease								
Cardiovascular diseases								
No	2851 (88.46)	1133 (86.23)	Reference	6499 (88.91)	2783 (86.51)	Reference		
Yes	372 (11.54)	181 (13.77)	1.00 (0.83–1.21)	811 (11.09)	434 (13.49)	1.30 (1.15–1.46)	0.02	81

*HR* hazard ratio adjusted for age, lifestyle factors, dental behavior, diabetes mellitus, and cardiovascular diseases.

*P* values from Cochran’s Q test < 0.1 or I^2^ from Higgin’s I^2^ test > 50% implied that there was a significant difference by the stratified factor.

## Discussion

The present study aimed to evaluate the bidirectional association between PD and DM, as well as that between PD and CVD, as defined by a physician, using information from both clinical reports and self-reports. After adjustment, our findings showed that PD was significantly associated with an increased risk of DM. Furthermore, we found a reciprocal relationship between PD and CVD, where each increased the risk of the other.

### Bidirectional association between periodontal disease and diabetes mellitus

Previous longitudinal studies have found evidence of the elevated risk of DM among individuals with PD^8–10^, supported by the results of previous meta-analyses that reached the same conclusion^6,7^. These reports are similar to the present findings, implying that PD is likely to increase the risk of DM by 22%. The most explored explanation for this relationship is the elevation of systemic pro-inflammatory cytokine markers following the occurrence of PD^15,16^. These markers include increased levels of tumour necrosis factor-alpha and interleukin-16, which subsequently cause insulin resistance directly by inducing C-reactive protein^15,16^.

Prior studies suggested that DM is a significant a risk factor for PD^8,13–15^, and some even reported a two-way relationship between the two diseases^6–8,13^. Here, we found a significant association before adjustment for CVD, implying that the association was not independent of the presence of CVD in this study population. However, in terms of direction and effect size, the results indicated that the risk of PD was higher among people with DM.

Only a few studies have evaluated this bidirectional association in the same study population^8,19^ and reported disparate results; Morita et al*.* found significant two-way associations between periodontal status and diabetes, while Alshihayb et al*.* did not. These differences in significance may be due to differences in the sample size because statistical significance may be influenced by the number of observations^36^. The first study included a much larger study population compared to the second; hence, the apparent significance. In terms of effect size, both studies showed similar results wherein the risk of the outcomes was increased (the HR values were more than 1). In this regard, our study had similar findings, wherein the effect sizes showed that PD and diabetes may increase each other’s risk of occurrence.

### Bidirectional association between periodontal disease and cardiovascular disease

The present findings are consistent with those of previous reports concluding that there is an increased risk of CVD among individuals with PD^12,13^. The risk of CVD in the present study increased by 27%, which is relatively similar to the increase of 20% indicated by a previous meta-analysis, although the latter’s definition of CVD included various comorbidities^12^. A similar impact of PD was also observed regarding the elevated risk of coronary heart disease^37^ and heart failure^38^, two diseases included in our definition of CVD.

In contrast to studies of PD as a risk factor for CVD, the number of studies evaluating the reverse association remains limited^12^. Our findings add to the evidence indicating that individuals with CVD are more likely to develop PD than those without CVD, implying the presence of a two-way relationship between these diseases that is independent of DM, with similar magnitudes for both associations. Stratified analysis of the increased risk of CVD revealed significance only among women. This may be due to differences in the effects of hormones on the periodontium in general and on the development of PD in particular^39,40^.

The strength of the present study lies in its use of a nationally representative sample linked to two other databases in Korea, thus allowing the inclusion of broader information as well as more variables in the analysis. However, this study has some limitations. First, the definition of PD was based only on the history of diagnosis by a physician, which may only apply to participants who visited a dentist and usually addressed problems regarding their oral condition. However, the exact time of disease onset or the first occurrence of the disease could not be tracked. Second, it was not possible to assess the role of medication or treatment, which might affect the association between exposure and outcome. For example, previous studies have consistently reported that periodontal treatment is associated with a better blood glucose value^41,42^, and the effect of DM on the risk of PD may also differ between individuals with controlled and uncontrolled diabetes^41,43^. Third, the associations of brushing frequency and the use of rinsing solution with the outcomes appeared to oppose prior reports and current guidelines for oral hygiene^44–46^. It should be noted that in this survey, dental behaviours were assessed based only on participants’ responses regarding whether they complied with a certain behaviour or not, although the survey was conducted before the diseases were reported by a physician. For example, the participants were only asked if they had used a rinsing solution the day before or not. Thus, the technical correctness and frequency of the behaviour could not be confirmed. Fourth, the roles of other oral parameters, such as the Community Periodontal Index and number of remaining teeth, could not be evaluated as data on these variables were not available. Further studies that use other definitions to ascertain PD and include more possible confounding variables in the analysis are recommended; these studies are needed to provide more evidence regarding the bidirectional association between PD and DM.

## Conclusion

In conclusion, our study found a bidirectional association between PD and CVD, independent of age, sex, lifestyle factors, BMI, dental behaviour, and DM. Furthermore, a positive association between PD and an increased risk of DM was found after adjusting for the same covariates. Regarding the reverse association, the effect size and direction suggested an increased risk of PD among participants with diabetes, although this was not statistically significant. Through this study, we clarified the bidirectional association between PD and systemic diseases among Korean people, in whose context this bidirectional relationship has not been reported in the same study population.

This study may be useful for promoting the prevention of PD, DM and CVD. In promoting oral health, people may be informed that by preventing PD, they will also lower their risk of developing systemic disease. The advantages of preventing PD may also be cited when promoting strategies for diabetes and CVD prevention. Furthermore, people with PD may benefit from suggestions to pay attention to their glucose levels as well as risk of CVD.

### Supplementary Information

Supplementary Information.

## Data Availability

The data used from the KNHANES study is available in the Korea Disease Control and Prevention Agency repository, https://knhanes.kdca.go.kr/knhanes/main.do. However, the data from HIRA and NHID, as well as the linked dataset from three databases are not publicly available and can only be accessed on request from Korea Health Information Service.

## Abbreviations

ALT: Alanine aminotransferase
AST: Aspartate transaminase
BMI: Body Mass Index
CI: Confidence intervals
DMFT: Decayed, missing, and filled teeth
FPG: Fasting plasma glucose
HIRA: Health Insurance Review and Assessment Service
HR: Hazard ratio
ICD: International Classification of Disease
KNHANES: Korean National Health Nutrition Survey
NHID: National Health Information Database
