# Supplementary material for: Bidirectional associations between periodontal disease and systemic diseases: a nationwide population-based study in Korea
Source: Sci Rep. 2023 Aug 28;13:14078. doi: 10.1038/s41598-023-41009-4 (PMC10462734; doi:10.1038/s41598-023-41009-4)
Supplement: Supplementary file 1 — Supplementary Information. [file 41598_2023_41009_MOESM1_ESM.pdf]

Supplementary Materials

Bidirectional Associations between Periodontal Disease and Systemic Diseases: A Nationwide Population-Based Study in Korea

Salma Nabila[1,2,*], Jaesung Choi[3,4*,], Ji-Eun Kim[1,2], Seokyung Hahn[5,6], In-Kyung Hwang[7], Tae-Il Kim[7], Hee-Kyung Park[8,**], and Ji-Yeob Choi[1,2,3,9,**]

[1]Department of Biomedical Sciences, Seoul National University Graduate School, Seoul, Korea
[2]BK21plus Biomedical Science Project, Seoul National University College of Medicine, Seoul, Korea
[3]Institute of Health Policy and Management, Seoul National University Medical Research Center, Seoul, Korea
[4]St. Jude Children's Research Hospital, Memphis, Tennessee, USA
[5]Division of Medical Statistics, Medical Research Collaborating Center, Seoul National University Hospital, Seoul, Korea
[6]Department of Medicine, Seoul National University College of Medicine, Seoul, Korea
[7]Department of Periodontology, Dental Research Institute, Seoul National University Dental Hospital, Seoul National University School of Dentistry, Seoul, Korea
[8]Department of Oral Medicine and Oral Diagnosis, Dental Research Institute, Seoul National University Dental Hospital, Seoul National University School of Dentistry, Seoul, Korea
[9]Cancer Research Institute, Seoul National University, Seoul, Korea

[*]These two authors are equal contributors to this work and designated as co-first authors.
[**]Co-corresponding authors: Hee-Kyung Park
Department of Oral Medicine and Oral Diagnosis
Seoul National University Dental Hospital
101 Daehak-ro, Jongno-gu, Seoul, Korea, 110-749
Tel: +82-2-2072-2610
E-mail: dentopark@snu.ac.kr

Ji-Yeob Choi
Department of Biomedical Sciences
Seoul National University Graduate School
103 Daehak-ro, Jongno-gu, Seoul, Korea, 110-799
Tel: +82-2-740-8922/Fax: +82-2-3673-2167
E-mail: jiyeob.choi@gmail.com

Supplementary Table 1. Disease identification criteria

Disease	ICD-10 Code	Identification criteria
Gingivitis	K05	• Provision of the relevant diagnostic code at least two times within 30 days, or<br>• There was information on one of the following health claim codes on the medical record:<br>1. Mild (U2240, U1010)<br>2. Moderate (U1020, U1051, U1052, U1071, U1072, U1081, U1082)<br>3. Severe (U4412, U4413, U4414)
Diabetes	E11-E14	• Was given diabetes medication after diagnosis, or<br>• Provision of the relevant code at least two times within a year, or<br>• Reported history of being diagnosed with diabetes by a physician
Cardiovascular diseases	I20-I25, I50	• Medical record of being hospitalized for at least two days because of the relevant disease code
Cancer	C00-C97	• Provision of the relevant code in the main and sub diagnosis at least two times within a year<br>• Medical record of being hospitalized because of the relevant disease code
Cerebrovascular diseases	G45-G46,<br>I60-I69	• Medical record of being hospitalized for at least two days because of the relevant disease code
Hypertension	I10-I13, I15	• Was given hypertension medication after diagnosis, or<br>• Provision of the relevant code at least two times within a year, or<br>• Reported history of being diagnosed with hypertension by a physician
Hyperlipidemia	E78	• Was given hyperlipidemia medication after diagnosis, or<br>• Provision of the relevant code at least two times within a year, or
Arthritis	M15-M19, M47, M05-M06	• Provision of the relevant code at least two times within a year<br>• Reported history of being diagnosed with arthritis or rheumatic by a physician
Osteoporosis	M80-81	• Provision of the relevant code at least two times within a year<br>• Reported history of being diagnosed with osteoporosis by a physician
Gastrointestinal diseases	K25-K31,<br>K50-52	• Provision of the relevant code at least two times within a year<br>• Reported history of being diagnosed with gastrointestinal diseases by a physician
Infectious diseases	A00-B99	• Participants who had a medical record of being diagnosed with the relevant code at least two times within a year
Tooth decay	K02, K04	• Provision of the relevant diagnostic code at least two times within 30 days and<br>• There was information on one of the following health claim codes on the medical record: U0001, U0002, U0010, U0020, U0050, U0060, U0074, U0101, U0116, U0121, U0126, U0210, U0131, U0132, U0133, U0134, U0135, U0136, U0137, U0138, U0151, U0152, U0153, U0154, U0200

Supplementary Table 2. Baseline characteristics and hazard ratio for diabetes mellitus

Variables	Total	Diabetes mellitus	
	N (%)	Event (%)	HR (95% CI)
	14523	1151 (7.93)	
Sociodemographic factors			
Sex			
Men	4452 (30.65)	398 (34.58)	Reference
Women	10071 (69.35)	753 (65.42)	1.01 (0.82–1.23)
Income level[1]			
<200	4754 (32.73)	538 (46.74)	Reference
200–399	4629 (31.87)	339 (29.45)	0.99 (0.85–1.16)
≥400	5140 (35.39)	274 (23.81)	0.82 (0.69–0.97)
Age			
20–39	4687 (32.27)	98 (8.51)	Reference
40–59	5564 (38.31)	455 (39.53)	3.42 (2.73–4.29)
≥60	4272 (29.42)	598 (51.95)	5.42 (4.28–6.86)
Residential area			
Urban	11279 (77.66)	844 (73.33)	Reference
Rural	3244 (22.34)	307 (26.67)	0.94 (0.82–1.09)
Lifestyle factors and BMI			
Smoking status			
Never smokers	9450 (67.79)	741 (66.40)	Reference
Former smokers	2611 (18.73)	212 (19.00)	0.80 (0.53–1.21)
Current smokers	1880 (13.49)	163 (14.61)	1.01 (0.67–1.50)
Smoking pack–year			
Never smokers	9450 (71.21)	741 (68.42)	Reference
Light smokers	2629 (19.81)	167 (15.42)	1.04 (0.69–1.55)
Moderate smokers	812 (6.12)	116 (10.71)	1.47 (0.97–2.24)
Heavy smokers	380 (2.86)	59 (5.45)	1.64 (1.03–2.62)
Missing	1252	68	
Alcohol consumption			
Non–drinker	4157 (29.86)	435 (39.08)	Reference
Drinker	9765 (70.14)	678 (60.92)	0.91 (0.80–1.05)
Physical activity			
No exercise	5530 (52.04)	529 (55.51)	Reference
<150mins/wk	1275 (12.00)	88 (9.23)	0.83 (0.65–1.05)
≥150mins/wk	3822 (35.96)	336 (35.26)	0.92 (0.80–1.07)
Missing	3896	198	
BMI (kg/m^2)			
<18.5	655 (4.52)	20 (1.74)	0.90 (0.54–1.49)
18.5–22.9	6310 (43.57)	317 (27.57)	Reference
23–24.9	3467 (23.94)	288 (25.04)	1.43 (1.20–1.70)
≥25	4051 (27.97)	525 (45.65)	2.35 (2.02–2.73)
Biomarkers[2]			
AST			
Normal	13057 (96.48)	1004 (93.05)	Reference
High	477 (3.52)	75 (6.95)	1.96 (1.52–2.53)
Missing	1039	72	
ALT			
Normal	12757 (94.61)	967 (89.62)	Reference
High	727 (5.39)	112 (10.38)	1.99 (1.61–2.46)
Missing	1039	72	
FPG			
<100	10434 (77.45)	455 (42.21)	Reference
100–125	2718 (20.18)	455 (42.21)	3.17 (2.75–3.66)
≥126	320 (2.38)	168 (15.58)	13.58 (11.13–16.56)
Missing	1051	73	
Cholesterol			

Variables	Total	Diabetes mellitus	
	N (%)	Event (%)	HR (95% CI)
<200	8622 (66.15)	575 (53.29)	Reference
200–239	3270 (25.09)	356 (32.99)	1.13 (0.98–1.30)
≥240	1142 (8.76)	148 (13.72)	1.44 (1.19–1.74)
Missing	1039	72	
Blood pressure			
Normal	2191 (17.28)	684 (64.47)	Reference
Pre–hypertension	5477 (43.20)	367 (34.59)	1.36 (1.19–1.57)
Hypertension	5010 (39.52)	10 (0.94)	1.41 (1.12–1.76)
Dental behavior			
Brushing frequency			
≤1	2191 (15.09)	215 (18.68)	Reference
2	5477 (37.71)	495 (43.01)	1.04 (0.86–1.25)
3	5010 (34.50)	336 (29.19)	1.00 (0.82–1.23)
≥4	1845 (12.70)	105 (9.12)	1.03 (0.80–1.34)
Dental floss			
No	11347 (81.09)	1009 (90.41)	Reference
Yes	2646 (18.91)	107 (9.59)	0.76 (0.62–0.94)
Interdental brush			
No	12093 (86.42)	109 (50.46)	Reference
Yes	1900 (13.58)	107 (49.54)	0.99 (0.81–1.23)
Mouth rinsing solution			
No	11764 (84.07)	947 (84.86)	Reference
Yes	2229 (15.93)	169 (15.14)	1.10 (0.93–1.31)
DMFT			
≤4	4723 (34.16)	390 (34.70)	Reference
8–5	4008 (28.99)	304 (27.05)	0.97 (0.83–1.13)
>8	5096 (36.86)	430 (38.26)	0.91 (0.78–1.05)
Disease history (yes versus no)			
Tooth decay	6147 (42.33)	573 (49.78)	1.35 (1.19–1.52)
Cardiovascular diseases	1590 (10.95)	281 (24.41)	1.73 (1.48–2.01)
Cancer	1760 (12.12)	215 (18.68)	1.22 (1.04–1.44)
Cerebrovascular diseases	546 (3.76)	81 (7.04)	1.09 (0.83–1.42)
Hypertension	4002 (27.56)	636 (55.26)	1.97 (1.69–2.30)
Hyperlipidemia	1878 (12.93)	315 (27.37)	1.75 (1.51–2.03)
Arthritis and rheumatic	4993 (34.38)	634 (55.08)	1.39 (1.20–1.60)
Osteoporosis	1542 (10.62)	219 (19.03)	1.26 (1.05–1.50)
Gastrointestinal	10452 (71.97)	956 (83.06)	1.64 (1.39–1.94)
Infectious diseases	7269 (50.05)	607 (52.74)	1.19 (1.05–1.35)

HR, hazard ratio adjusted for age, sex, lifestyle factors, BMI, baseline FPG level, and history of cardiovascular disease; BMI, body mass index; AST, aspartate transaminase; ALT, alanine transaminase; FPG, fasting blood glucose; DMFT, Decayed, Missing, and Filled Teeth index.

[1] Income is in Korean 10.000 won;

[2] AST and ALT levels were considered high if the value was >40 IU/L; blood pressure was grouped into normal (systolic < 120 and diastolic < 80 mmHg), pre-hypertension (systolic 120–139 and diastolic 80–89 mmHg), and hypertension (systolic ≥ 140 or diastolic ≥ 90 mmHg).

Supplementary Table 3. Baseline characteristics and hazard ratio for cardiovascular diseases

Variables	Total	Cardiovascular diseases	
	N (%)	Event (%)	HR (95% CI)
	14315	509 (3.56)	
Sociodemographic factors			
Sex			
Men	4457 (31.14)	178 (34.97)	Reference
Women	9858 (68.86)	331 (65.03)	1.23 (0.88–1.73)
Income level[1]			
<200	4595 (32.10)	285 (35.99)	Reference
200–399	4572 (31.94)	117 (22.99)	0.89 (0.69–1.15)
≥400	5148 (35.96)	107 (21.02)	0.87 (0.66–1.14)
Age			
20–39	4718 (32.96)	19 (3.73)	Reference
40–59	5583 (39.00)	141 (27.70)	5.50 (3.38–8.94)
≥60	4014 (28.04)	349 (68.57)	15.02 (9.24–24.40)
Residential area			
Urban	11183 (78.12)	343 (67.39)	Reference
Rural	3132 (21.88)	166 (32.61)	1.06 (0.85–1.33)
Lifestyle factors and BMI			
Smoking status			
Never smokers	9234 (67.18)	311 (63.73)	Reference
Former smokers	2560 (18.62)	116 (23.77)	1.00 (0.48–2.10)
Current smokers	1951 (14.19)	61 (12.50)	0.87 (0.42–1.79)
Smoking pack–year			
Never smokers	9234 (70.79)	311 (65.47)	Reference
Light smokers	2602 (19.95)	85 (17.89)	1.14 (0.56–2.33)
Moderate smokers	822 (6.30)	56 (11.79)	1.62 (0.78–3.36)
Heavy smokers	386 (2.96)	23 (4.84)	0.96 (0.41–2.26)
Missing	1271	34	
Alcohol consumption			
Non–drinker	4019 (29.28)	214 (43.85)	Reference
Drinker	9705 (70.72)	274 (56.15)	0.97 (0.77–1.21)
Physical activity			
No exercise	5422 (51.98)	257 (57.88)	Reference
<150mins/wk	1244 (11.93)	38 (8.56)	1.02 (0.70–1.47)
≥150mins/wk	3765 (36.09)	149 (33.56)	1.08 (0.86–1.36)
Missing	3884	65	
BMI (kg/m^2)			
<18.5	646 (4.53)	6 (1.18)	0.48 (0.15–1.52)
18.5–22.9	6235 (43.68)	159 (31.24)	Reference
23–24.9	3445 (24.14)	135 (26.52)	1.24 (0.94–1.63)
≥25	3947 (27.65)	209 (41.06)	2.00 (1.57–2.55)
Biomarkers[2]			
AST			
Normal	12915 (96.81)	434 (95.59)	Reference
High	425 (3.19)	20 (4.41)	0.76 (0.41–1.39)
Missing	975	55	
ALT			
Normal	12593 (94.40)	426 (93.83)	Reference
High	747 (5.60)	28 (6.17)	0.99 (0.63–1.54)
Missing	975	55	
FPG			
<100	9855 (73.94)	247 (54.65)	Reference
100–125	2692 (20.20)	142 (31.42)	1.27 (0.99–1.63)
≥126	782 (5.87)	63 (13.94)	1.35 (0.93–1.97)
Missing	986	57	
Cholesterol			

Variables	Total N (%)	Cardiovascular diseases Event (%)	HR (95% CI)
<200	8578 (64.30)	277 (61.01)	Reference
200–239	3652 (27.38)	128 (28.19)	0.88 (0.70–1.12)
≥240	1110 (8.32)	49 (10.79)	0.91 (0.64–1.30)
Missing	975	55	
Blood pressure			
Normal	10833 (75.91)	286 (56.30)	Reference
Pre–hypertension	2838 (19.89)	155 (30.51)	1.62 (1.28–2.03)
Hypertension	600 (4.20)	67 (13.19)	3.08 (2.29–4.16)
Dental behavior			
Brushing frequency			
≤1	2185 (15.26)	119 (23.38)	Reference
2	5365 (37.48)	218 (42.83)	1.00 (0.76–1.34)
3	4918 (34.36)	134 (26.33)	0.87 (0.64–1.20)
≥4	1847 (12.90)	38 (7.47)	0.90 (0.58–1.38)
Dental floss			
No	11152 (80.91)	450 (92.02)	Reference
Yes	2632 (19.09)	39 (7.98)	0.90 (0.64–1.27)
Interdental brush			
No	11904 (86.36)	460 (94.07)	Reference
Yes	1880 (13.64)	29 (5.93)	0.75 (0.50–1.13)
Mouth rinsing solution			
No	11593 (84.10)	433 (88.55)	Reference
Yes	2191 (15.90)	56 (11.45)	1.06 (0.78–1.42)
DMFT			
≤4	4680 (34.35)	152 (30.46)	Reference
8–5	3966 (29.11)	124 (24.85)	0.95 (0.73–1.24)
>8	4980 (36.55)	223 (44.69)	0.96 (0.75–1.23)
Missing			
Disease history (yes versus no)			
Tooth decay	6014 (42.01)	235 (46.17)	1.30 (1.06–1.60)
Diabetes mellitus	1382 (9.65)	132 (25.93)	1.84 (1.44–2.35)
Cancer	1649 (11.52)	109 (21.41)	1.42 (1.10–1.83)
Cerebrovascular diseases	409 (2.86)	40 (7.86)	1.40 (0.92–2.13)
Hypertension	3384 (23.64)	351 (68.96)	4.31 (3.37–5.50)
Hyperlipidemia	1850 (12.92)	118 (23.18)	1.33 (1.04–1.69)
Arthritis and rheumatic	4677 (32.67)	322 (63.26)	1.94 (1.54–2.45)
Osteoporosis	1372 (9.58)	126 (24.75)	1.74 (1.34–2.26)
Gastrointestinal	10161 (70.98)	425 (83.50)	1.87 (1.42–2.47)
Infectious diseases	7086 (49.50)	253 (49.71)	1.14 (0.93–1.40)

HR, hazard ratio adjusted for age, sex, lifestyle factors, BMI, and history of diabetes mellitus; BMI, body mass index; AST, aspartate transaminase; ALT, alanine transaminase; FPG, fasting blood glucose; DMFT, Decayed, Missing, and Filled Teeth index.

[1]Income is in Korean 10.000 won;

[2]AST and ALT levels were considered high if the value was >40 IU/L; blood pressure was grouped into normal (systolic < 120 and diastolic < 80 mmHg), pre-hypertension (systolic 120–139 and diastolic 80–89 mmHg), and hypertension (systolic ≥ 140 or diastolic ≥ 90 mmHg).

Supplementary Table 4. Association between BMI and periodontal disease, diabetes mellitus, and cardiovascular disease

BMI (kg/m²)	Dataset 1			Dataset 2			Dataset 3		
	Total N (%)	Periodontal disease		Total N (%)	Diabetes mellitus		Total N (%)	Cardiovascular disease	
		Events N (%)	HR[1] (95% CI)		Events N (%)	HR[2] (95% CI)		Events N (%)	HR[3] (95% CI)
<18.5	487 (3.97)	168 (3.63)	0.95 (0.81-1.12)	655 (4.52)	20 (1.74)	0.95 (0.57-1.58)	646 (4.53)	6 (0.27)	0.47 (0.15-1.47)
18.5-22.9	4524 (36.88)	1909 (41.29)	Reference	6310 (43.57)	317 (27.57)	Reference	6235 (43.68)	159 (7.03)	Reference
23-24.9	4524 (36.88)	1120 (24.23)	1.14 (1.06-1.24)	3467 (23.94)	288 (25.04)	1.24 (1.04-1.48)	3445 (24.14)	135 (5.97)	1.25 (0.95-1.65)
25-29.9	2423 (19.75)	1298 (28.08)	1.19 (1.11-1.28)	3673 (25.36)	458 (39.83)	1.65 (1.41-1.94)	3568 (25.00)	1947 (86.07)	2.00 (1.57-2.56)
≥ 30	310 (2.53)	128 (2.77)	1.13 (0.95-1.36)	378 (2.61)	67 (5.83)	1.98 (1.48-2.64)	379 (2.66)	15 (0.66)	2.09 (1.21-3.60)
<18.5	487 (4.64)	168 (3.71)	0.95 (0.81-1.12)	655 (4.52)	20 (1.74)	0.95 (0.57-1.58)	646 (4.53)	6 (1.18)	0.47 (0.15-1.47)
18.5-22.9	4524 (43.10)	1809 (40.00)	Reference	6310 (43.57)	317 (27.57)	Reference	6235 (43.68)	159 (31.24)	Reference
23-24.9	2423 (23.08)	1120 (24.76)	1.14 (1.06-1.23)	3467 (23.94)	288 (25.04)	1.24 (1.04-1.48)	3445 (24.14)	135 (26.52)	1.25 (0.95-1.65)
25-27.4	1967 (18.74)	930 (20.56)	1.19 (1.09-1.29)	2660 (18.37)	310 (26.96)	1.55 (1.31-1.84)	2603 (18.24)	143 (28.09)	1.90 (1.46-2.48)
≥27.5	1095 (10.43)	496 (10.97)	1.18 (1.07-1.31)	1391 (9.60)	215 (18.70)	1.94 (1.61-2.35)	1344 (9.42)	66 (12.97)	2.24 (1.64-3.07)

[1]Hazard ratio adjusted for age, sex, and dental behavior.
[2]Hazard ratio adjusted for age, sex, lifestyle factors, BMI, baseline FPG level, and history of cardiovascular disease.
[3]Hazard ratio adjusted for age, sex, lifestyle factors, BMI, and history of diabetes mellitus.

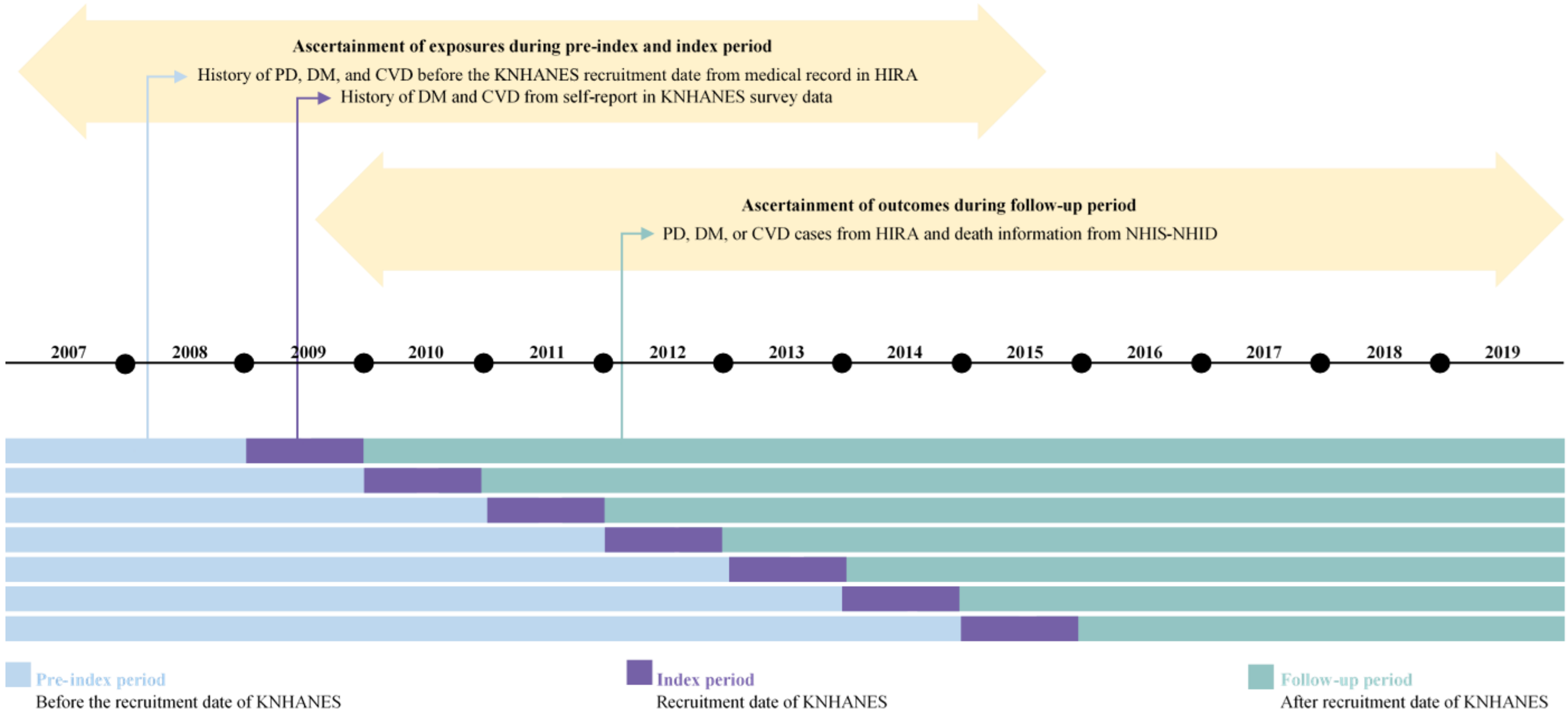

Supplementary Figure 1. Study scheme indicating pre-index, index, and follow-up period
